# Differences in Encoding Strategy as a Potential Explanation for Age-Related Decline in Place Recognition Ability

**DOI:** 10.3389/fpsyg.2020.02182

**Published:** 2020-09-10

**Authors:** Christopher Hilton, Veronica Muffato, Timothy J. Slattery, Sebastien Miellet, Jan Wiener

**Affiliations:** ^1^Psychology Department, Ageing and Dementia Research Centre, Bournemouth University, Bournemouth, United Kingdom; ^2^Biological Psychology and Neuroergonomics, Berlin Institute of Technology, Berlin, Germany; ^3^Department of General Psychology, University of Padua, Padua, Italy; ^4^Active Vision Lab, School of Psychology, University of Wollongong, Wollongong, NSW, Australia

**Keywords:** ageing, place recognition, visual attention, eye-tracking, object-location binding, perspective taking

## Abstract

The ability to recognise places is known to deteriorate with advancing age. In this study, we investigated the contribution of age-related changes in spatial encoding strategies to declining place recognition ability. We recorded eye movements while younger and older adults completed a place recognition task first described by Muffato et al. (2019). Participants first learned places, which were defined by an array of four objects, and then decided whether the next place they were shown was the same or different to the one they learned. Places could be shown from the same spatial perspective as during learning or from a shifted perspective (30° or 60°). Places that were different to those during learning were changed either by substituting an object in the place with a novel object or by swapping the locations of two objects. We replicated the findings of Muffato et al. (2019) showing that sensitivity to detect changes in a place declined with advancing age and declined when the spatial perspective was shifted. Additionally, older adults were particularly impaired on trials in which object locations were swapped; however, they were not differentially affected by perspective changes compared to younger adults. During place encoding, older adults produced more fixations and saccades, shorter fixation durations, and spent less time looking at objects compared to younger adults. Further, we present an analysis of gaze chaining, designed to capture spatio-temporal aspects of gaze behaviour. The chaining measure was a significant predictor of place recognition performance. We found significant differences between age groups on the chaining measure and argue that these differences in gaze behaviour are indicative of differences in encoding strategy between age groups. In summary, we report a direct replication of Muffato et al. (2019) and provide evidence for age-related differences in spatial encoding strategies, which are related to place recognition performance.

## Introduction

Knowing where you are in the world is vital to many fundamental daily tasks. Such orientation begins with recognising the place you are in. Recognising a place from a known viewpoint can be achieved by matching stored images of that place with current visual input. However, we often must recognise places from a viewpoint, which is different from when we first learnt the place. In this case, we must additionally engage spatial perspective taking mechanisms to resolve the difference in perspective between our representation of that place and the current viewpoint.

To successfully recognise a place, it must be distinguished from those that are similar. Humans encounter many places, which share common object features, for example, road signs, traffic lights, or trees. Thus, there are many cases in which recognising the individual object identities alone is not sufficient for successful place recognition. To distinguish a place from those that are similar, object identity information must be supplemented with information about the arrangement of the objects in space (Pertzov et al., 2012). As such, place encoding and recognition are complex tasks requiring the binding of object identities to their spatial locations (object-location binding) integrated with the ability to retrieve these representations from a different perspective (spatial perspective taking).

Muffato et al. (2019) investigated how the mechanisms underlying place recognition are affected by ageing. In their experiment, participants first experienced an encoding phase during which they were shown an image of a place to learn. In the subsequent test phase, participants were shown a different image for which they had to decide whether the depicted place was the same or different to the place shown in the encoding phase. The places in their experiment were made up by an array of four unique objects. To test different mechanisms involved in place recognition, places in the test phase could be manipulated in several ways as follows.

Object identity memory was tested in the substitute condition in which one object in the place was replaced with a novel object between encoding and test phase. In this condition, the recognition performance of older adults was similar to that of younger adults, suggesting that memory for the objects in a place is preserved with advancing age. This result is in line with other spatial learning experiments (Cushman et al., 2008; Head and Isom, 2010; Allison and Head, 2017) and suggests that age-related deficits in place recognition ability are not simply driven by an inability of older adults to remember object identities. Object-location binding was tested in the swap condition during which the same objects were presented in the test place as in the encoding place, but with the spatial positions of two objects swapped. Participants would have only recognised the change in spatial arrangement if object-location binding was successful (c.f. Pertzov et al., 2012). Muffato et al. (2019) found that older adults’ recognition performance was particularly affected by the swap changes. This finding suggests that object-location binding mechanisms are impaired in older adults (see Dai et al., 2018).

Muffato et al. (2019) also tested spatial perspective taking ability. In their experiment, test places could be shown from either the same or from a different perspective to that during encoding. Recognition performance declined with the introduction of a perspective shift, but this decline was similar for both age groups. This finding is consistent with previous research, which suggests that spatial perspective taking ability is not affected by cognitive ageing (Watanabe, 2011; Watanabe and Takamatsu, 2014). The picture is mixed however, with other studies reporting an age-related decline in spatial perspective taking ability (Inagaki et al., 2002; Montefinese et al., 2015).

Current explanations for age-related changes in place recognition ability focus on the neurodegeneration of the hippocampal circuit (see Klencklen et al., 2012; Li and King, 2019). The hippocampus is involved in the development of viewpoint independent spatial representations and in spatial perspective taking (King et al., 2002; Hartley et al., 2007; Hartley and Harlow, 2012). Further, object location binding mechanisms are also thought to be hippocampus dependent (Piekema et al., 2006; Postma et al., 2008). Given the age-related neurodegeneration of the hippocampus, which underpins place recognition mechanisms, it is unsurprising that older adults are impaired in place recognition ability. What remains unclear is the nature of the link between hippocampal decline and place recognition impairment. Older adults could simply be attempting to use the same mechanisms as younger adults, with recognition impairment resulting from sub-optimal execution due to hippocampal decline. This explanation would account for the object-location binding deficits in older adults, but conflicts with the findings of Muffato et al. (2019) showing preserved spatial perspective taking ability in older age. An alternative explanation is that ageing may be accompanied by a shift in place learning and recognition strategies in order to compensate for hippocampal decline (Gutchess et al., 2005; Zhong and Moffat, 2018). These compensatory strategies may be less effective for successful place recognition. Muffato et al. (2019) highlighted that they were unable to discriminate age-related differences in place encoding strategies as a potential explanation for decline in place recognition ability. We address this point in the current study, in which we present a replication of the task used in Muffato et al. (2019), with the addition of eye-tracking to record gaze behaviour.

Eye-tracking is an established method to investigate the mechanisms and strategies involved in solving cognitive tasks. Already, early eye movement research demonstrated that gaze patterns in response to a visual stimulus changed depending on the task to be performed (Yarbus and Levy-Schoen, 1968). In fact, eye movements can be considered as even more than just an artefact of cognitive processes, but an integral part of these processes. This view was well-summarised by Neisser (1967), who argued that recall of visual information is a reconstruction process, involving coordination of visual memory and eye movements rather than simple retrieval of stored pictures. More recent work supports this conception, showing that the relationship between the scan-path displayed when learning an image and later recalling an image predict accuracy of recall (Laeng and Teodorescu, 2002). Moreover, this replay of eye movements is accompanied by image-specific patterns of brain activity during recall (Bone et al., 2019). Indeed, eye-tracking has been used to investigate strategies in many cognitive domains, such as learning (for a review, see Lai et al., 2013), reading (for a review, see Rayner, 1998), memory (for a review, see Hannula et al., 2010), face recognition (Chaby et al., 2017), and navigation (Mueller et al., 2008; Livingstone-Lee et al., 2011; Andersen et al., 2012). This link between eye movements and cognition extends to the solving of spatial tasks (Thomas and Lleras, 2007).

Older adults display eye movement patterns different to that of younger adults in a range of tasks. During route learning, older adults spend less time encoding landmarks, which contribute to an increased likelihood to become disoriented on subsequent attempts to traverse that route (Grzeschik et al., 2019). Age-related differences are also apparent in basic gaze parameters such as reduced saccade amplitudes and increased fixation durations (Dowiasch et al., 2015) as well as in various scan-path measures, such as in reading, where older adults skip more words than younger adults. This is known as the risky reader strategy (Rayner et al., 2006), which in turn leads to more regressions in text than younger adults (McGowan and Reichle, 2018). Paterson et al. (2013) demonstrated that these differences in eye movements during reading are not a result of impaired oculomotor control, which is preserved with age, but are driven by changes in reading strategy. Age-related changes in strategy use are also apparent when remembering the position of 2D objects on a screen, where older adults have been shown to rely on fixation reinstatement to a greater extent than younger adults (Wynn et al., 2018). Fixation reinstatement is the process of reapplying eye movements to the relevant screen locations which objects were shown in and has been suggested to be a strategy used to support memory (Olsen et al., 2014). While there is not a universal method to characterise gaze scan-paths (see Anderson et al., 2014), various implementations such as those discussed here demonstrate that spatio-temporal measures of gaze behaviour provide an insight into differences between age groups.

It is not always the case, however, that age effects are observed in eye movements. Hilton et al. (2019) had younger and older participants learn a route through a complex virtual environment while recording eye movements. Although they observed age-related differences in route learning ability consistent with other studies (e.g., Head and Isom, 2010; Wiener et al., 2012), they did not find differences between older and younger adults on a range of eye movement measures. This is consistent with the notion of preserved oculomotor control in ageing (Paterson et al., 2013) as well as other accounts of age-equivalence of eye movement patterns in the absence of a task driven strategy differences (Pratt et al., 1997, 2006; Abrams et al., 1998). The existing research demonstrates that age-related differences in strategy use can be reflected in differences in gaze parameters, various scan-path measures, and dwell time on relevant stimuli. Conversely, in situations where older and younger adults use the same cognitive strategies to solve a task, similar gaze behaviour across age groups can be expected.

In the present experiment, we used eye-tracking to study if the age-related difference in place recognition ability reported by Muffato et al. (2019) was the result of different place encoding strategies. We expected to replicate behavioural results from their study. That is, we expected (1) older adults to perform worse than younger adults overall and (2) for age to interact with condition. Specifically, we expected a greater performance deficit for older adults in the swap condition in which object locations were swapped in the place as compared to the substitute condition in which an object was replaced with a novel object. If any observed age-related differences were to be a result of maladaptive encoding strategy use by older adults, we expected to also find differences in gaze behaviour during place encoding. Specifically, we analysed eye movement parameters (c.f. Dowiasch et al., 2015; Hilton et al., 2019) and dwell time on task-relevant regions of interest (ROI; c.f. Grzeschik et al., 2019). Finally, we introduce a novel scan-path measure, which captures spatio-temporal characteristics of gaze behaviour. On all the measures listed above, we report not only age group comparisons but also the extent to which gaze behaviour relates with performance to explore how they are relevant in the context of spatial learning.

## Materials and Methods

### Participants

Thirty young and 32 older participants took part in the experiment. Older participants were screened for cognitive impairment using the Montreal Cognitive Assessment (MoCA; Nasreddine et al., 2005), and three participants were excluded from the data using a cut-off score of 23 (Luis et al., 2009; Waldron-Perrine and Axelrod, 2012). Table 1 summarises the demographic data of the final participant groups. Ethical approval was granted by Bournemouth University Research Ethics Panel, and written informed consent was gained from all participants who participated in exchange for either course credits or monetary compensation for their time.

**Table 1:** Participant demographics.

	Sex		Age		MoCA	
		*n*	Mean	*SD*	Mean	*SD*
Younger	Female	15	21.07	3.28		
	Male	15	22.00	5.53		
Older	Female	16	71.31	5.77	27.88	1.67
	Male	13	76.54	6.51	26.85	1.86

In the study conducted by Muffato et al. (2019), participants were split into three age groups; 20–29, 60–69, and 70–79 years old. In their study, the object-location binding deficit was observed between the 20–29 and 60–69 age groups, but no additional decline was observed between the 60–69 and 70–79 age groups. Since the aim of the present study was to investigate the age-related object-location binding deficit in place recognition, which did not change between the two groups of older adults in Muffato et al. (2019), we grouped all our participants over the age of 65 into one older adult participant group.

### Design

There were three independent variables in this experiment which were age group (younger and older), perspective shift (0°, 30°, and 60°), and place manipulation (same, swap, and substitute). The behavioural dependent variable was sensitivity (*d*’) to detect a place change, which was calculated from the response data. There were also several eye-tracking dependent variables, which are presented in the eye-tracking section of the methods.

**Figure 1:**
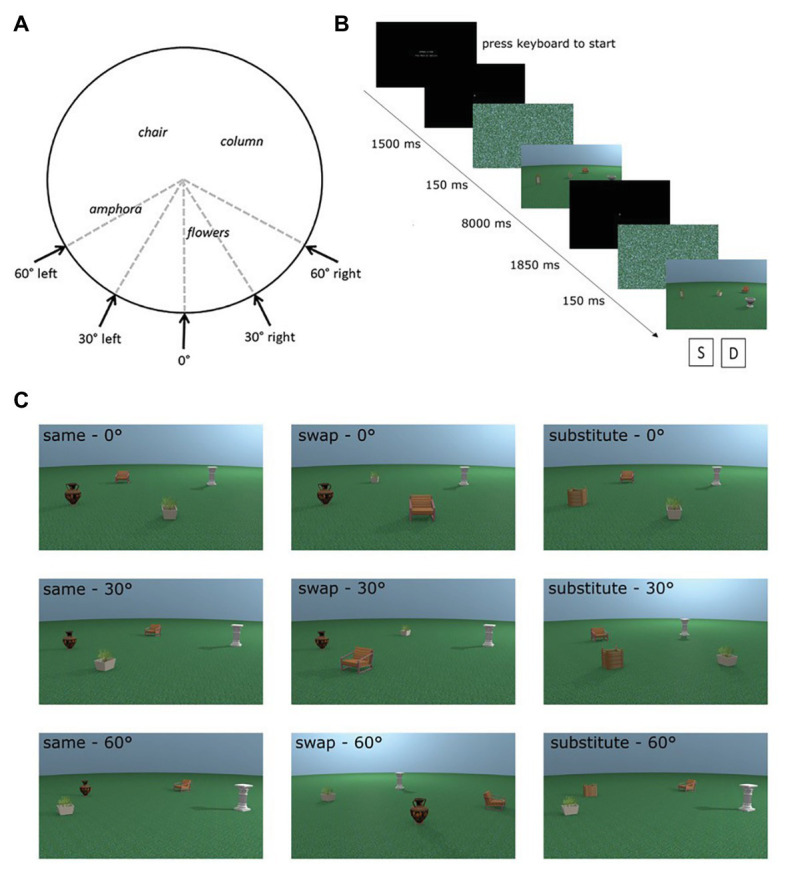
Adapted from Muffato et al. (2019). **(A)** Overhead schematic of the different possible viewpoints a test place could be shown from. Encoding places were always shown from the 0° viewpoint. **(B)** Sequence of a trial in the experiment. **(C)** Examples of all possible test conditions for one encoding place incorporating manipulation (swap or substitute) and perspective shift (0°, 30°, and 60°).

We used eight different places in the encoding phases of the experiment. For each place, test images were rendered from the same viewpoint as the encoding stimulus and at 30° and 60° perspective shifts. The direction of the perspective shift was counterbalanced to occur equally in the left and the right directions (see Figure 1A). Additional images of test places were rendered from all perspectives with an object replaced for a novel object (substitute condition) or with two objects swapped in space (swap condition; see Figure 1C for examples of each test condition). For more detail about the creation of the stimuli, see Muffato et al. (2019).

We made one change in the experiment design from the study conducted by Muffato et al. (2019). In their experiment, a black and white mask was displayed before each stimulus in order to disrupt any after-images from the previous stimulus. In the present study, we changed the mask to a scrambled version of one of the places in the experiment. This change was made to ensure visual consistency between the mask and the stimulus presented in the trial in terms of colour, luminosity, etc., so as not to introduce artefacts into the eye-tracking data, such as changes in pupil dilation at the beginning of each trial.

### Materials

OpenSesame 3.1.4 (Mathôt et al., 2012) was used to display the stimuli and collect responses, with the PyGaze plug-in for eye-tracking recording. The experiment was presented on a 102 cm screen (diagonal) with an aspect ratio of 16:9 and a resolution of 1,920 × 1,080 pixels. Participants sat 1 m away from the screen and responded to the task using the X and M keys on the keyboard, which were labelled as S (same) and D (different), respectively. Eye movements were recorded using an Eyelink II (SR Research) head-mounted eye-tracker at a rate of 500 Hz. Calibration used a nine-point grid, and an online drift correction was performed before every trial. Large drift errors initiated a recalibration before continuing the experiment.

### Procedure

Each trial comprised an encoding and test phase. During the encoding phase, participants were shown an image of a place for a fixed time of 8 s and were instructed to learn the depicted place. In the subsequent test phase, participants were shown the image of the test place. Participants had to indicate whether the test place was identical or different from the encoding place. Participants were carefully instructed that a place could be the same even if it was presented from a different perspective in the test phase. Figure 1B details the exact trial procedure and timings of the different phases of the trial. There were a total of 72 trials consisting of eight trials for each of the nine conditions [three place manipulations (same, swap, and substitute) × 3 perspective shifts (0°, 30°, and 60°)]. The trials were in three blocks, which were presented in a random order, with trials from each condition evenly distributed across the three blocks.

### Eye-Tracking Analysis

We restricted the analysis of the eye movement data to the encoding phase for two reasons. First, as described above, our research question focused on potential differences in visual encoding strategies. Second, response times and therefore quantity of eye-tracking data in the test phase varied widely between participants, with many participants producing a little as one or two fixations during the test phase trials. Since older adults produced longer response times than younger adults, age comparisons of eye movement data in the test phase would have been heavily confounded by differences between age groups in the amount of eye-tracking data. This was not an issue in the encoding phase, which had a fixed duration of 8 s.

**Figure 2:**
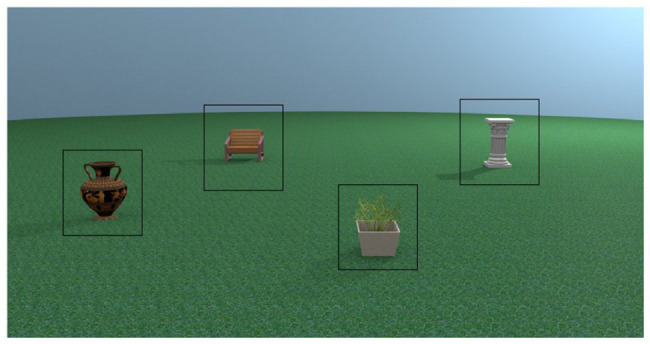
Example region of interest (ROI) placement for one learning stimulus.

Given the lack of previous work utilising eye-tracking methodology in place recognition paradigms, we performed several exploratory analyses on the gaze data in this experiment. For each analysis, we first investigated if there was an age difference in the measure, and then whether that measure was predictive of place recognition performance. For analyses which focused on location of gaze, we used ROI. Each object had an identically sized ROI (see Figure 2 for example ROI placement), and the rest of the stimulus was considered as a non-object ROI for a total of five ROIs per stimulus. The same ROI templates were used in each analysis which required them.

First, we analysed dwell time on objects in the place compared to the background. On this measure, Grzeschik et al. (2019) reported that older adults spent less time than younger adults looking at objects placed at intersections during a navigation task. Therefore, we might expect that older adults would also look at objects less than younger adults in our task. On the other hand, our environment was very sparse compared to that used in Grzeschik et al. (2019), with no distinguishable features to draw attention other than the objects, and thus we were unsure as to whether this finding would replicate in the present experiment. Next, we analysed fixation and saccade parameters as a descriptive insight into the oculomotor behaviour displayed in the different participant groups.

While the analyses described above gives a descriptive insight into gaze behaviour, they are limited in terms of assessing encoding strategies as these measures do not capture the spatio-temporal characteristics of the gaze behaviour during encoding. As discussed earlier, eye movements between features in the environment are an integral part of the encoding system, and the order in which environmental features are looked at could provide insight into specific encoding strategies and differences in encoding strategies between age groups. To capture the order in which objects in the place were looked at during encoding, we developed a gaze measure which will be referred to as *chaining*.

#### Chaining

**Figure 3:**
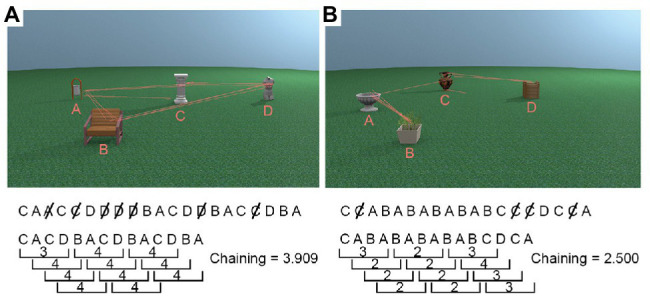
Example chaining calculations. First, duplicates were removed from the sequence of objects gazed at, and then the number of unique objects in every window of four for the whole trial was averaged to produce the chaining measure. **(A)** A high chaining trial in which participants’ gaze was repeatedly directed towards objects which were not recently looked at. **(B)** A low chaining trial in which participants’ gaze moved back and forth between the same two objects for a large portion of the trial.

For each trial, we first recorded the order in which the five interest areas (four objects + non-object background) were visited, discarding successive fixations within the same ROI. Fixations on the non-object background ROI were also removed as it did not contain any task relevant information to be processed, leaving only the sequence in which participants viewed the four object ROIs^1^. Once we obtained a vector with the order in which the four object ROIs were looked at during encoding, we used a sliding window with a size of four (reflecting the maximum possible chain of four unique objects) to calculate how many unique objects (i.e., ROIs) were looked at. This window moved through the vector, and we calculated the chaining measure, i.e., the average number of ROIs participants looked at for every four ROI transitions during encoding (Figure 3 visualises the chaining measure in detail). The maximum value of the chaining measure is 4, and the minimum value is 2. High chaining values represent encoding strategies in which participants’ “chained” all objects together in a sequence and repeatedly looked at them in the same order (see Figure 3A). Low chaining values, in contrast, represent trials during which gaze shifted between the same subsets of available objects before moving on to newer objects, for example, switching back and forth between two objects (see Figure 3B).

## Results

We analysed the data using linear mixed effects (LMEs) models and generalised linear mixed effects (GLMEs) models in (R Core Team, 2019) using the lme4 package (version 1.1-21; Bates et al., 2015). For each model, we began with an intercept only model and iteratively added by-participant and by-item slopes. The final model was selected based on AIC comparison between models.

### Behavioural

#### Sensitivity

Accuracy data were converted into *d*-prime scores [*d*’ = *z*(false alarm rate) − *z*(hit rate)] for each participants’ responses for every condition, which represent their ability to detect a change in the stimulus. We ran an LME on *d*’ with fixed effects of manipulation (sum contrast coding: swap and substitute), perspective (successive differences contrasts: 0°, 30°, and 60°), and age group (sum contrast coding: younger and older). We included participant as a random effect. Since *d*’ scores are calculated across trials, item could not be included as a random effect in this model. The final model included by-participant perspective and condition slopes and was the same as in Muffato et al. (2019). Coefficients, standard errors, and *t*-values are reported in Table 2.

**Table 2:** Linear mixed effect (LME) model for *d*’ scores.

Predictors	*d* Prime			Replication of Muffato et al. (2019)
	Estimates	std. Error	*t*-value	
**Intercept**	**2.11**	**0.09**	**24.80** ^*^	
**Manipulation**	**−0.15**	**0.03**	**−4.84** ^*^	Yes
**Age group**	**0.36**	**0.09**	**4.19** ^*^	Yes
**Perspective (0° vs. 30°)**	**−0.40**	**0.09**	**−4.31** ^*^	Yes
Perspective (30° vs. 60°)	−0.15	0.08	−1.76	No
Manipulation * age group	**0.07**	**0.03**	**2.29** ^*^	Yes
Manipulation * perspective (0° vs. 30°)	−0.06	0.05	−1.12	Yes
Manipulation * perspective (30° vs. 60°)	0.01	0.05	0.19	Yes
Age group * perspective (0°–30°)	0.05	0.09	0.54	Yes
Age group * perspective (30°–60°)	−0.01	0.08	−0.16	Yes
Age group * manipulation * perspective (0°–30°)	0.06	0.05	1.24	Yes
Age group * manipulation * perspective (30°–60°)	**−0.11**	**0.05**	**−2.22** ^*^	No

* Significant *t* values (|*t*| > 1.96); highlighted in bold.

There were effects of age group, manipulation, and perspective. Specifically, younger participants had significantly higher *d*’ than older participants, *d*’ was significantly lower in the swap condition than the substitute condition, and *d*’ was significantly lower for a 30° perspective shift compared to a 0° perspective shift. There was no significant effect of perspective shift between 30° and 60° on *d*’ scores.

**Figure 4:**
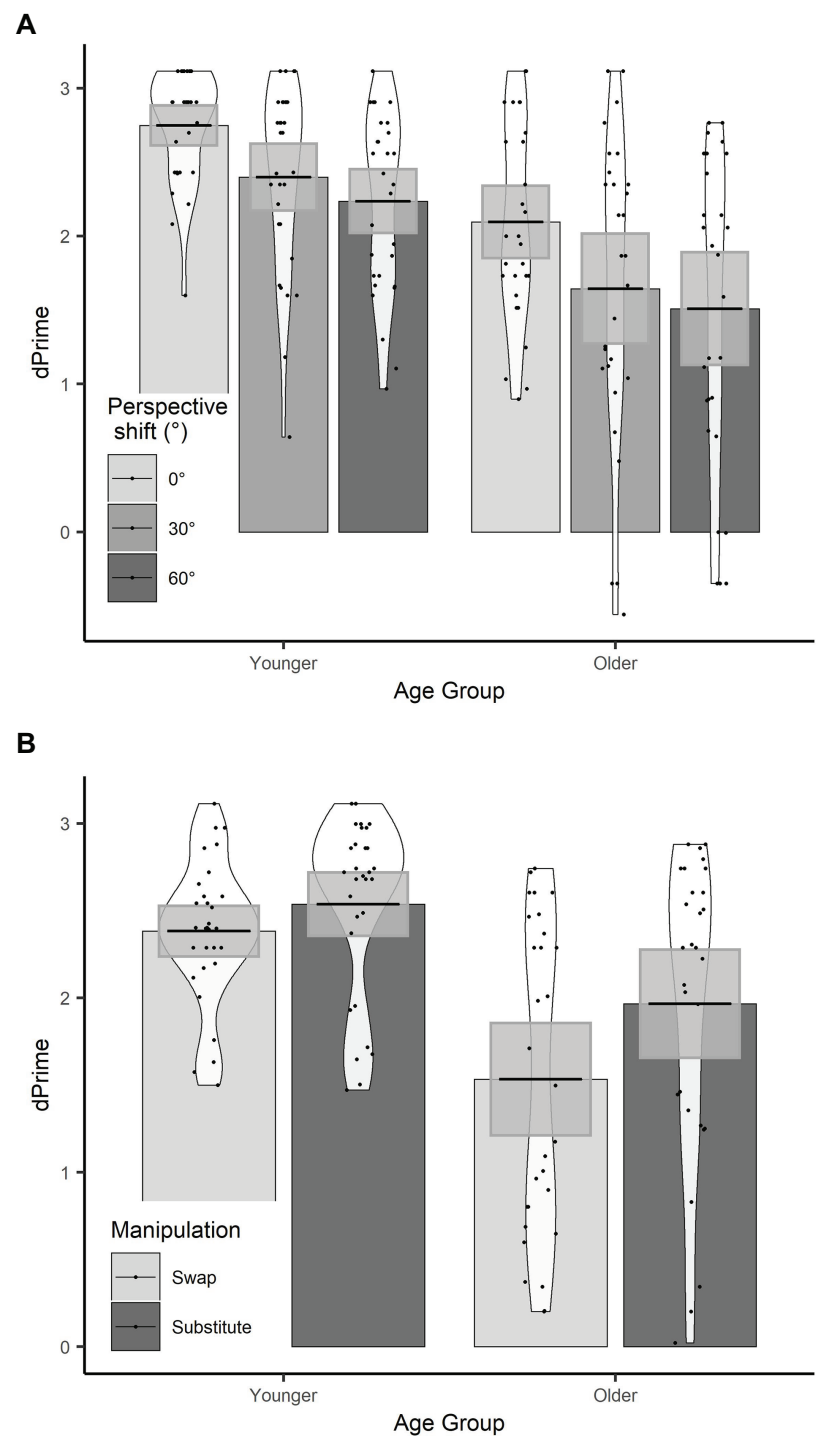
**(A)** *d*’ scores for age group × perspective; **(B)** *d*’ scores for age group × manipulation. Plots show mean averages with confidence interval error bars, individual data points, and density profiles.

There was a manipulation by age group interaction, which showed that the decline in *d*’ in the swap compared to the substitute condition was greater for the older adults compared to the younger adults (see Figure 4). There was also a three-way age group by manipulation and perspective (30°–60°) interaction, which shows that the effect of perspective shift (30° vs. 60°) for older adults in the swap condition, and younger adults in the substitute condition was smaller than for older adults in the substitute condition and younger adults in the swap condition. When the data were split by manipulation and models were run separately for the swap and the substitute conditions, there was no significant two-way age groups by perspective (30° vs. 60°) interaction in either model (substitute: *β* = −0.10, *SE* = 0.10, *t* = 1.05; swap: *β* = −0.13, *SE* = 0.11, *t* = −1.19).

### Eye-Tracking

#### Pre-processing

Eye movements were parsed using SR Research algorithms. We filtered out eye movements, which fell outside of the screen boundaries or contained a blink. We also removed the first fixation of every trial since this was likely an artefact of the pre-trial fixation cross in the centre of the screen. Saccades which exceeded the maximum amplitude (41.35°va) or velocity (1,500°/s) that should be possible based on distance of the participant from the screen, and screen size were regarded as tracker error and were removed. An LME with the fixed effect of age group (sum contrast coding; younger and older) and random factors of participant and item (intercept only) showed no significant differences in the amount of eye-tracking data removed (out of 8,000 ms) between older (mean = 526.72 ms) and younger (mean = 576.76 ms) age groups (*β* = 25.02, *SE* = 28.91, *t* = 0.87).

#### Time Spent Looking at Objects

An LME with the fixed effect of age group (sum contrast coding: younger and older) and random factors of participant and item (intercept only) revealed that fixations on the objects represented a greater proportion of the encoding phase for younger adults compared to older adults (mean = 0.76; *β* = 0.03, *SE* = 0.01, *t* = 2.61)^2^.

To investigate whether differences in time spent looking at objects during encoding contributed to the difference in place recognition performance, we conducted a GLME on trial performance (binomial; correct or incorrect). Fixed effects were proportion of time spent looking at objects (continuous and centred), age group (sum contrast coding: younger and older), manipulation (sum contrast coding: same, swap, or substitute), and random factors of participant and item (intercept only). Time spent looking at objects did not predict trial performance (*β* = −0.02, *SE* = 0.07, *z* = −0.25, *p* = 0.799) and did not interact with condition (swap: *β* = 0.07, *SE* = 0.08, *z* = 0.90, *p* = 0.369; substitute: *β* = −0.07, *SE* = 0.09, *z* = −0.79, *p* = 0.433) or with age group (*β* = −0.03, *SE* = 0.07, *z* = −0.49, *p* = 0.624).

#### Parameters

We conducted an LME model for each gaze parameter with age group as a fixed effect (sum contrast coding: younger and older) and random factors of participant and item (intercept only). Coefficients, standard errors, and *t*-values are reported in Table 3. In summary, older adults produced more fixations, with shorter durations. This was accompanied by more saccades executed by older adults, which did not differ from younger adults in terms of amplitude and velocity.

**Table 3:** Means for each age group and separate LME model results for each gaze parameter.

Parameter	Younger group mean	Older group mean	Estimates	std. Error	*t*-value
Saccade amplitude (°va)	7.72	7.60	0.06	0.14	0.41
Saccade peak velocity (°/s)	265.07	266.81	−0.87	5.51	−0.16
Saccade Avg. velocity (°/s)	150.20	146.32	1.94	2.44	0.80
**Saccade frequency (/s)**	**2.83**	**3.03**	**−0.10**	**0.05**	**−1.97** ^*^
Saccade duration (ms)	42.36	42.96	−0.30	0.64	−0.47
**Saccade sum duration (ms)**	**894.35**	**979.96**	**−42.80**	**21.72**	**−1.97** ^*^
**Saccade quantity**	**21.18**	**22.76**	**−0.79**	**0.40**	**−1.97** ^*^
**Fixation duration (ms)**	**303.14**	**274.19**	**14.48**	**5.52**	**2.62** ^*^
**Fixation frequency (/s)**	**2.89**	**3.12**	**−0.12**	**0.04**	**−2.65** ^*^
**Fixation quantity**	**22.73**	**24.36**	**−0.81**	**0.35**	**−2.30** ^*^
Fixation sum duration (ms)	6,528.89	6,493.32	17.79	30.16	0.59

* Significant *t* values (|*t*| > 1.96); highlighted in bold.

To investigate whether gaze parameter profiles predicted performance, we conducted a GLME^3^ on trial accuracy (binomial; correct or incorrect) with a selection of gaze parameters as fixed effects. Where multiple parameters can be considered as highly related measures, only one was selected (for example, number of fixations and fixation frequency are high correlated when trial length is fixed, *r* = 0.99). Number of fixations, average fixation duration, saccade amplitude, and saccade average velocity were included as fixed effects (all centred). Participant and item were included in the model as random effects (intercept only). Coefficients, standard errors, and *z*-values are reported in Table 4 and show that patterns of fixation and saccade parameters did not predict trial accuracy.

**Table 4:** Generalised linear mixed effect (GLME) model for gaze parameters and trial accuracy.

Predictors	Accuracy			
	Estimates	std. Error	*z*-value	*P*
**(Intercept)**	**1.90**	**0.13**	**14.67**	**<0.001** ^*^
Number of fixations	−0.03	0.10	−0.32	0.748
Average fixation duration	−0.02	0.10	−0.20	0.840
Average saccade amplitude	0.01	0.13	0.05	0.963
Average saccade velocity	−0.03	0.13	−0.25	0.805

* Significant *z* values (|*z*| > 1.96); highlighted in bold.

#### Chaining

**Figure 5:**
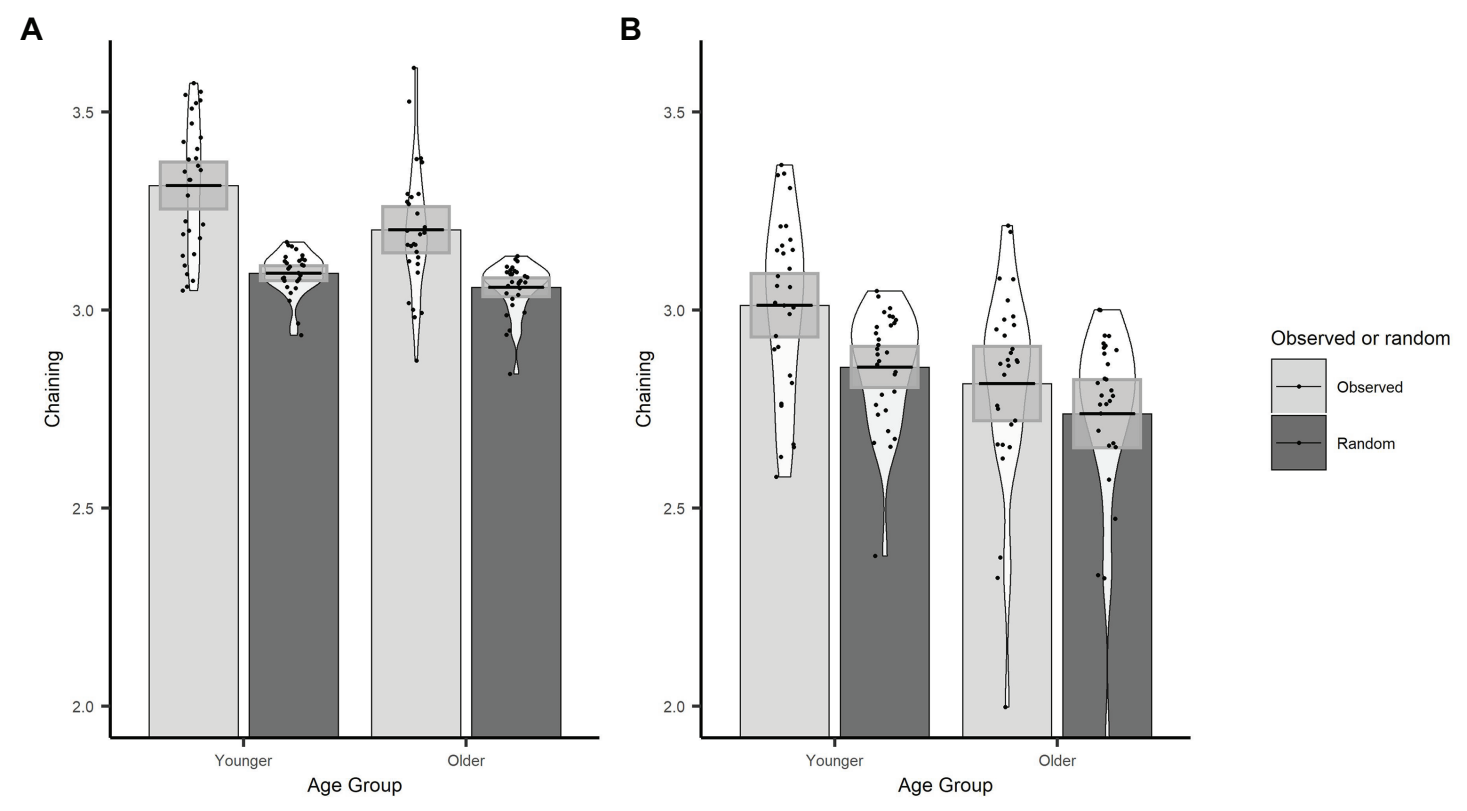
Observed and randomized chaining across age groups with fixations on non-object ROIs removed (A) or included (B). Plots show mean averages with confidence interval error bars, individual data points, and density profiles.

In order to demonstrate that the chaining measure captures the extent to which gaze behaviour was actively controlled through the use of a cognitive strategy, we first compared our observed chaining values to those that would occur if gaze was randomly directed between objects. To calculate the chaining value for random gaze behaviour, we randomised the order of the observed ROI vectors for every trial. Here, we used the actual data, which preserve the number of visits to each ROI, and the only change is to the order in which those ROIs were visited through the trial. We then conducted an LME on chaining values with data source (sum contrast coding: observed and random) and age group (sum contrast coding: younger and older) as fixed effects and random factors of participant and item (intercept only). The model revealed that chaining values were larger for the observed data than the random data (*β* = 0.09, *SE* < 0.01, *t* = 30.84) and that this interacted with age (*β* = 0.02, *SE* < 0.01, *t* = 6.35). To follow up the interaction, we conducted separate models for younger and older groups which showed that observed chaining values were larger than random values for both the younger (*β* = 0.11, *SE* < 0.01, *t* = 25.97) and the older (*β* = 0.07, *SE* < 0.01, *t* = 17.62) age group (see Figure 5A); however, the effect was larger for the younger adults which explains the interaction.

Next, we used an LME model to investigate age differences in chaining behaviour. Age group was included as a fixed effect (sum contrast coding: younger and older), and participant and item were included as random factors (intercept only). The model revealed that younger adults had higher chaining values than older adults (*β* = 0.06, *SE* = 0.02, *t* = 2.64).

To assess whether chaining behaviour was related to task performance, we conducted a GLME on trial accuracy (binomial; correct or incorrect) with chaining value (continuous and centred), age group (sum contrast coding: younger and older), and condition (sum contrast coding: same, swap, and substitute) as fixed effects, and participant and item as random factors (intercept only). The model revealed that higher chaining behaviour in the encoding phase predicted better recognition performance in the test phase (*β* = 0.15, *SE* = 0.06, *z* = 2.71, *p* = 0.007). This effect did not interact with age group (*β* = 0.04, *SE* = 0.05, *z* = 0.73, *p* = 0.464) or condition (swap: *β* = 0.02, *SE* = 0.07, *z* = 0.36, *p* = 0.718; substitute: *β* = 0.07, *SE* = 0.08, *z* = 1.00, *p* = 0.319).

For the above analysis of chaining, non-object ROI visits were removed (see Methods section “Eye-tracking analysis”). Since we report above that older adults spent a larger proportion of the encoding phase looking at non-object ROIs, we recalculated the chaining measure, including non-object ROIs. Non-object ROIs were not counted as unique interest areas but as disruptions. For example, if there were three unique objects visited and one visit to the non-object ROI within a window of four ROI visits, the chaining measure would be 3. This decision was consistent with our original point that non-object ROIs offered no information which would aid place learning, and thus may be considered as a disruption to efficient encoding strategies.

We conducted the same set of analyses as for the original chaining measure implementation. As before, the observed chaining values differed from random chaining values (*β* = 0.06, *SE* < 0.01, *t* = 15.34), which was the case for both older (*β* = 0.04, *SE* = 0.01, *t* = 6.99) and younger participants (*β* = 0.08, *SE* = 0.01, *t* = 14.87). Observed chaining values were still higher for younger participants than for older participants (*β* = 0.10, *SE* = 0.03, *t* = 3.15; see Figure 5B). However, this version of the chaining measure did not predict trial accuracy (*β* = 0.01, *SE* = 0.06, *z* = 0.24, *p* = 0.809).

## Discussion

In this experiment, we used eye-tracking and a place recognition task to investigate age-related changes in place encoding strategies. Participants were shown a place during the encoding phase and then had to decide whether the place was identical or had changed in a subsequent test phase. We replicated behavioural findings reported by Muffato et al. (2019). Older adults performed worse than younger adults, particularly when object positions were swapped, a manipulation that required object-location binding to solve. Recognition ability was worse when a spatial perspective shift was introduced, but older adults were not differentially affected by perspective shifts compared to younger adults. We found small age-effects in several gaze parameters and in the time spent looking at objects; however, these gaze measures did not correlate with place recognition performance. We also developed a new gaze measure, chaining, which captures spatio-temporal aspects of gaze behaviour. The chaining measure was different between age groups and one variant of this new measure was related to recognition performance.

As we expected, overall sensitivity to detect changes in the places was lower for older adults than for younger adults. This result is consistent with many other accounts of age-related decline in spatial learning abilities (for reviews, see Moffat, 2009; Klencklen et al., 2012; Lester et al., 2017). We also found that sensitivity to detect changes in the place was lower overall for the swap condition in which two of the four objects were exchanged between encoding and test than for the substitute condition in which one object was replaced with a new object. The increased difficulty of the swap condition can be explained by the requirement to engage object location binding mechanisms to successfully recognise the place (Postma et al., 2008; Pertzov et al., 2012), whereas the substitute condition can be solved with object identity knowledge alone. Importantly, an interaction between age group and manipulation revealed that the drop in sensitivity between substitute and swap conditions was greater for older adults than for younger adults. Given that older adults perform better in the substitute condition than the swap condition, this deficit cannot be explained by a lack of object identity knowledge, which appears to remain relatively intact in our adults (c.f. Cushman et al., 2008; Head and Isom, 2010). Instead, they can be explained by a specific age-related decline in object-location binding. These findings are consistent with other accounts of age-related decline in object location binding ability (e.g., Dai et al., 2018).

In our study, we found that the initiation of a perspective shift was associated with a drop in recognition sensitivity, which did not interact with age as reported by Muffato et al. (2019). This can be explained by the initiation of additional spatial perspective taking mechanisms, which are not active in the 0° condition, therefore incurring additional cognitive load (Holmes et al., 2018). In contrast to Muffato et al. (2019), we did not find that sensitivity continued to drop with increasing degrees of perspective change (30° vs. 60°). This could be a reflection of additional perspective shifts being less costly, due to the fact that spatial perspective taking mechanisms are already engaged in the 30° condition, and thus no new mechanisms need to be engaged to solve the 60° condition. Indeed, the 0°–30° shift effect in Muffato et al. (2019) was more than double the size of the 30°–60° shift. In our study, the 30°–60° perspective shift effect did not reach significance, although our *t*-value was close (*t* = 1.76). Given that our study had fewer participants than Muffato et al. (2019), and the relatively small size of their 30°–60° perspective shift effect, we may have lacked power to detect this effect. An alternative explanation is that increase in perspective change may not increase the difficulty of place recognition; however, this interpretation seems unlikely since it conflicts with other evidence showing that increasing perspective changes are associated with reduced recognition performance (Diwadkar and McNamara, 1997; Montefinese et al., 2015).

The behavioural results of this study are a direct replication of those found by Muffato et al. (2019). As such, the main results are that older adults are impaired in object-location binding dependent place recognition but have preserved perspective taking ability. The novel contribution of the present study was to investigate the contribution of place encoding strategies to the age-related impairment observed in place recognition ability.

We recorded gaze behaviour to assess differences in place encoding strategies between younger and older adults. We found differences between age groups in several gaze parameters. Specifically, older adults produced more fixations in the 8-s encoding phase than younger adults, with shorter average fixation duration. Accordingly, they performed more saccades than younger adults, but saccade amplitudes and velocity were comparable between age groups. These findings conflict with those of Dowiasch et al. (2015) who reported that older adults made fewer saccades which had lower amplitudes than those performed by younger adults while locomoting through a real-world environment. Açik et al. (2010) also reported lower saccade amplitudes in older adults while viewing a complex visual image, although their results show higher saccade frequency and reduced fixation durations, which are consistent with our results. Açik et al. (2010) suggested that because their task did not contain a recall memory element, older adults were able to employ an efficient image exploration strategy, which involved performing a series of short saccades and fixations throughout the image. The same argument is true for the study conducted by Dowiasch et al. (2015), although their study contains a locomotion element which could be responsible for the increased fixation time, since older adults are known to alter their gaze behaviour in an attempt to avoid falling (for a review, see Uiga et al., 2015).

Indeed, in paradigms which do contain a memory element, such as visual search tasks where items in the stimulus must be compared to a target object in memory, the reverse pattern of gaze behaviour is observed. Older adults fixate more often and for longer durations (Williams et al., 2009) likely due to older adults being more cautious about accepting or rejecting items as targets (Porter et al., 2010). In our task, the encoding phase did not require participants to compare the stimulus to visual memory traces, which could explain why we observed reduced fixation durations and increased saccade frequency as found by Açik et al. (2010). Equivalence of saccade amplitudes and velocities between our age groups may be a result of the simple stimuli used, which is in contrast to the visually dense stimuli used in the study by Açik et al. (2010). With only four objects presented against a visually simple background, there are limited choices as to where gaze should be directed, and since older and younger adults viewed the same stimuli, eye movements between these objects would produce saccades of similar amplitudes. Following this, increased frequency of saccades between objects, with shorter fixation times on those objects could be a result of differences in encoding strategy between age groups.

In our study, gaze parameters were not predictive of place recognition performance. The coefficients we report from our models of gaze parameters reveal that the age differences are very small (for example, on the scale of less than one saccade and fixation per trial). In addition, gaze parameters are not independent of each other, and visual encoding strategies are likely reflected in a combination of these parameters as scan-paths performed throughout the trial, which forms a part of the memory trace for that place (Bone et al., 2019). In this case, we would expect that breaking those scan-paths into their component parts (gaze parameters), which also removes any temporal element in the data, would also reduce the predictive power of eye movements for recognition performance. Combined with the small effect sizes we observe between age groups, it is not surprising that individual parameters of oculomotor behaviour did not correlate with performance. To address this point and to gain a deeper understanding of visual encoding strategies, we developed the chaining measure.

The chaining measure was designed to quantify the extent to which participants are using an encoding strategy, which involves chaining multiple unique objects together during encoding. High chaining values represent an encoding strategy in which participants were likely to direct their gaze to an object which had not recently been inspected, creating a sequence of eye movements which bind (or chain) several different objects together. The chaining value is lowered when an object which has recently been attended to is revisited as opposed to gaze being directed towards a novel object.

We found that observed chaining was significantly higher than what would be expected if participants’ gaze transitioned randomly between objects. The difference between observed and random chaining suggests that the measure captured strategy directed gaze behaviour. The older adults in our study chained significantly less than younger adults during encoding. This finding is consistent with accounts of age-related changes in strategy in other cognitive tasks, such as during reading, where older adults are more likely to make regressions to previously read text than younger adults, likely as a result of skipping words (Rayner et al., 2006). The tendency to under-process important task relevant information is also present during route navigation in which older adults spend less time looking at landmarks (Grzeschik et al., 2019). In our study, we also found that older adults spent significantly less time looking at landmarks overall, alongside a reduction in individual fixation durations. In this scenario, regressions to recently attended objects to correct incidences of under-processing would have resulted in the lower chaining values. Such regressive saccades would be of similar amplitude and velocity as saccades to other objects in the place, which is consistent with our findings regarding these parameters.

One explanation for the reduced chaining patterns in our older adults could be age-related changes in working memory. It is well-established that several aspects of working memory change with advancing age (Klencklen et al., 2017; D’Antuono et al., 2020). Poorer visual working memory skills for older adults result in worse retention of visual features (Brockmole and Logie, 2013) and could be why the older adults in our study were more likely to re-fixate recently viewed objects, in order to refresh their representation. The decline in working memory span has been shown to extend beyond the visual domain, with general span deficits occurring in older age (Bopp and Verhaeghen, 2005). Bopp and Verhaeghen (2005) note in their meta-analysis that age differences in working memory span become apparent around list sizes of 4, which was the maximum possible chain size in our study. Bopp and Verhaeghen (2005) further report that age differences in span increase proportionally with increasing set sizes. If working memory span is an influencing factor in the chaining behaviour observed in our study, then more complex places with a larger quantity of objects (>4), as is common in the real world, may be even more difficult for older adults to encode.

We found that the initial implementation of the chaining measure (excluding fixations on the non-object background ROI) did predict recognition performance, which suggests that differences in visual encoding strategies contributed to the age-related place recognition deficit. There was no interaction between chaining value and condition when predicting performance, indicating that high chaining is an encoding strategy that is suited for both the substitute and the swap condition. This is not surprising, considering that the substitute condition can be solved with landmark identity information alone (as soon as an object is identified as novel the place can be accepted as different). Thus, any visual encoding strategy that is efficient to solve the swap condition would also be suited for the substitute condition. This is because the object-location binding which is required when solving swap trials also requires object identity knowledge (Pertzov et al., 2012). Given that older adults’ performance was less impaired in the substitute condition than the swap condition, their visual encoding strategy is likely to be somewhat efficient for the encoding of object identity. However, higher chaining behaviour as seen in our younger participants is better still for object identity encoding, since they outperform our older participants in the substitute condition. The reduced likelihood for older adults to sequence multiple objects together through their eye movements (lower chaining) may contribute to weaker spatial integration of the object arrangement, resulting in the additional difficulty that older adults experienced detecting the change in the swap condition.

Optimal chaining behaviour would result in a stereotyped fixation sequence with gaze being directed to the four objects repeatedly in the same order. Specifically, at the end of the object chain, gaze should return to the object in which the chain began to create a circular sequence (e.g., as shown in the example scan-path in Figure 3A). When the place does not change, the order of objects is the same, even if the viewpoint has changed provided the optimal chain is initiated from the same object. If two object positions were swapped however, the order would be disrupted, and the place can be identified as different. In this way, a temporal structure of the place is created through eye movements (Rucci et al., 2018; Heuer and Rolfs, 2019), where a swap of object locations results in a swap along the temporal dimension. Usually such temporal encoding of space is evident when stimuli dynamically appear and disappear or are highlighted, in a sequence (De Lillo et al., 2016) and has its own independent contribution to memory from concurrently formed spatial representations (Heuer and Rolfs, 2019).

If such viewpoint independent temporal structures were derived during place learning through gaze chaining, the need for perspective taking mechanisms would be circumvented, and thus could account for the lack of interaction between age and perspective shift in recognition performance in the present study as well as in Muffato et al. (2019). It is possible that in the current and earlier studies (e.g., Watanabe, 2011; Watanabe and Takamatsu, 2014; Muffato et al., 2019), participants were able to extract temporal information as an alternative to perspective taking. This may not be the case for studies which found that spatial perspective shifts do differentially affect performance for different age groups (e.g., Montefinese et al., 2015). If such an explanation is accurate, it is unlikely that participants are relying solely on any temporal representation of the place since we did find an overall main effect of perspective shift, and thus are more likely to be used in combination with spatial information (Heuer and Rolfs, 2019). Further research would be required to reconcile the role of temporal and spatial reference frames when solving spatial perspective changes and how this is affected by age.

We also reported a subsequent calculation of the chaining measure in which we included fixations to non-objects (background). Fixations on the non-object ROI were counted as disruptions, and thus the presence of non-object fixations in the scan-path reduced the chaining measure. In this implementation of the measure, the age effect increased in size, reflecting the increased likelihood of older adults to disrupt their chains with fixations away from the relevant objects. However, chaining did not correlate with recognition performance anymore. This is contrary to what one might expect given that there is no information in the non-object interest areas, which could aid the resolution of the task. Thus, eye movements to these non-object regions or background should have disrupted spatial encoding (Thomas and Lleras, 2007). If this was the case, we expected the chaining measure that included non-object fixations to have a larger correlation with recognition performance; however, we actually found the opposite. One explanation for the lack of association between chaining and performance using this version of the chaining measure is that while non-object fixations have not aided in solving the task, they were also not costly. Given that there is no complex visual information to be processed in the non-object regions, fixations in these areas may not have negatively affected the spatial representations of the place. This argument is supported by the finding that the time spent fixating at non-object regions also did not predict performance. Indeed, Shih et al. (2012) reported differences between age groups on time spent looking at objects in a spatial encoding task, and they also conclude that such object-oriented viewing does not promote memory about the general layout of the objects in space. Given this explanation, the inclusion of non-object fixations in the chaining measure served only to add noise to the data, and thus impacted on its predictive power.

If non-object fixations in our task were neutral with regards to place encoding, then why did older adults fixate non-object regions significantly more often than younger adults? This could be a result of reduced oculomotor accuracy in saccade landing sites for older adults (Sharpe and Zackon, 1987). If this were true however, we would also have expected lower average saccade amplitudes in older adults resulting from corrective saccades, which we did not find. Alternatively, visits to non-object regions may be an artefact of older adults attempting to rely on cues external to the object array. Indeed, older adults have been shown to rely more on geometric cues in the environment as opposed to objects or landmarks when orienting in space (Bécu et al., 2019). Further, current work from Segen et al. (2020) found that eye movements during place encoding were more exploratory in older adults than in younger adults. Segen et al. (2020) suggest that older adults rely on distal environmental cues to aid spatial encoding, more so than younger adults. In our task, there were no external environmental cues such as distal objects (Segen et al., 2020) or geometric features (Bécu et al., 2019), and so attempts from older adults to fixate on extra-object cues would have been futile.

In summary, we provide further evidence for age-related impairments in place recognition ability, particularly when recognition requires the use of object-location binding mechanisms. We show differences between age groups on several measures of eye movements, including chaining of objects through gaze. We explore how these differences could be indicative of differences in place encoding strategy and provide some first insights into the nature of these strategies.

## Data Availability Statement

The datasets presented in this study can be found in the Open Science Framework online repository: https://osf.io/6r7w5.

## Ethics Statement

The studies involving human participants were reviewed and approved by Bournemouth University Ethics Committee. The patients/participants provided their written informed consent to participate in this study.

## Author Contributions

CH, VM, and JW conceptualised the study. CH and VM implemented the experimental protocol, and CH collected the data. All authors provided input to data analysis protocols and interpretation of results. CH wrote the manuscript draft. VM, TS, SM, and JW reviewed the manuscript and provided input throughout the process. All authors contributed to the article and approved the submitted version.

### Conflict of Interest

The authors declare that the research was conducted in the absence of any commercial or financial relationships that could be construed as a potential conflict of interest.

## Footnotes

^1^In the first application of this measure, we also removed fixations, which occurred in the non-object ROI since our stimuli did not feature any relevant cues in non-object ROIs that could be used to solve the task. We later also report the chaining measure without removing non-object ROIs included in a follow-up analysis.

^2^Due to limitations of using LME analysis with proportion data, we checked this analysis using an LME on log transformed fixation duration on objects with the same fixed and random effects structure. The result was the same as the presented model on proportion of fixation time.

^3^Age group was omitted from the model due to issues with convergence.
